# IgG4 Autoantibodies in Organ-Specific Autoimmunopathies: Reviewing Class Switching, Antibody-Producing Cells, and Specific Immunotherapies

**DOI:** 10.3389/fimmu.2022.834342

**Published:** 2022-03-24

**Authors:** Inga Koneczny, John Tzartos, Marina Mané-Damas, Vuslat Yilmaz, Maartje G. Huijbers, Konstantinos Lazaridis, Romana Höftberger, Erdem Tüzün, Pilar Martinez-Martinez, Socrates Tzartos, Frank Leypoldt

**Affiliations:** ^1^ Division of Neuropathology and Neurochemistry, Department of Neurology, Medical University of Vienna, Vienna, Austria; ^2^ Neuroimmunology, Tzartos NeuroDiagnostics, Athens, Greece; ^3^ 2nd Department of Neurology, “Attikon” University Hospital, School of Medicine, National and Kapodistrian University of Athens, Athens, Greece; ^4^ Research Group Neuroinflammation and Autoimmunity, Department of Psychiatry and Neuropsychology, Faculty of Health, Medicine and Life Sciences, Maastricht University, Maastricht, Netherlands; ^5^ Department of Neuroscience, Aziz Sancar Institute of Experimental Medicine, Istanbul University, Istanbul, Turkey; ^6^ Department of Human Genetics, Leiden University Medical Center, Leiden, Netherlands; ^7^ Department of Neurology, Leiden University Medical Center, Leiden, Netherlands; ^8^ Department of Immunology, Laboratory of Immunology, Hellenic Pasteur Institute, Athens, Greece; ^9^ Department of Neurobiology, Hellenic Pasteur Institute, Athens, Greece; ^10^ Neuroimmunology, Institute of Clinical Chemistry and Department of Neurology, UKSH Kiel/Lübeck, Kiel University, Kiel, Germany

**Keywords:** IgG4 autoimmune disease, MHC, autoimmunity, IL-4, IL-10, Fab-arm exchange, memory B cells

## Abstract

Organ-specific autoimmunity is often characterized by autoantibodies targeting proteins expressed in the affected tissue. A subgroup of autoimmunopathies has recently emerged that is characterized by predominant autoantibodies of the IgG4 subclass (IgG4-autoimmune diseases; IgG4-AID). This group includes pemphigus vulgaris, thrombotic thrombocytopenic purpura, subtypes of autoimmune encephalitis, inflammatory neuropathies, myasthenia gravis and membranous nephropathy. Although the associated autoantibodies target specific antigens in different organs and thus cause diverse syndromes and diseases, they share surprising similarities in genetic predisposition, disease mechanisms, clinical course and response to therapies. IgG4-AID appear to be distinct from another group of rare immune diseases associated with IgG4, which are the IgG4-related diseases (IgG4-RLD), such as IgG4-related which have distinct clinical and serological properties and are not characterized by antigen-specific IgG4. Importantly, IgG4-AID differ significantly from diseases associated with IgG1 autoantibodies targeting the same organ. This may be due to the unique functional characteristics of IgG4 autoantibodies (e.g. anti-inflammatory and functionally monovalent) that affect how the antibodies cause disease, and the differential response to immunotherapies of the IgG4 producing B cells/plasmablasts. These clinical and pathophysiological clues give important insight in the immunopathogenesis of IgG4-AID. Understanding IgG4 immunobiology is a key step towards the development of novel, IgG4 specific treatments. In this review we therefore summarize current knowledge on IgG4 regulation, the relevance of class switching in the context of health and disease, describe the cellular mechanisms involved in IgG4 production and provide an overview of treatment responses in IgG4-AID.

## Introduction

IgG4 autoimmune diseases (IgG4-AID) are an emerging group of autoimmune diseases caused by IgG4 subclass autoantibodies (1, 2). Autoantibodies are key pathogenic players in many autoimmunopathies, and cause disease by a range of different effector mechanisms, such as complement activation, antibody-dependent cellular cytotoxicity or cross-linking and endocytosis of antigen (3). These are pathogenic mechanisms that depend on the antibody Fc region of IgG1-3 subclass antibodies, such as acetylcholine receptor (AChR) antibodies in myasthenia gravis, NMDAR antibodies in autoimmune encephalitis or of antibodies against aquaporin 4 (AQP4) in neuromyelitis optica. These effector functions are not available to the IgG4 subclass which has a structurally distinct Fc region (4, 5). IgG4 is produced in response to prolonged or strong antigen stimulation, and thought to play a role as anti-inflammatory or tolerogenic antibody (discussed in more detail below). Despite IgG4’s protective and tolerogenic role, in recent years two groups of rare diseases emerged, in which IgG4 takes the center stage: IgG4-AID that are caused directly by pathogenic IgG4 autoantibodies [such as MuSK myasthenia gravis (6, 7) or pemphigus vulgaris (8, 9), reviewed in more detail here (10)] and IgG4 related diseases [IgG4-RLD, such as IgG4-related pancreatitis (11) or IgG4-related periaortitis/periarteritis (12)]. IgG4-RLD are a rare multiorgan diseases that are characterized by tissue-destructive fibrotic lesions with lymphocyte and IgG4 plasma cell infiltrates and elevated serum IgG4 concentrations (13). Both IgG4-AID and IgG4-RLD are rare diseases of the immune system that may affect different target organs that are nevertheless most likely two unrelated groups of diseases (14). A key difference lies in the role of the IgG4 antibodies: In IgG4-RLD, increased serum concentrations of IgG4 are present in a majority of patients (15), but the antigenic targets of IgG4 and the role of IgG4 antibodies for pathogenicity is unclear [reviewed in detail here (16, 17)]. In contrast, IgG4-AID patients do not show the 10-fold increase in serum IgG4 levels as IgG4-RD patients (14), which otherwise– even though causing diverse symptoms and affecting different organs - all share one common feature: They are associated with antigen-specific, pathogenic IgG4 autoantibodies (1, 10).

Antibodies involved in IgG4-AID, known to date, target antigens in mainly four organ systems: 1) the central and peripheral nervous system with diseases such as MuSK myasthenia gravis (MG), anti-LGI1 and anti-Caspr2 encephalitis, anti-IgLON5 disease or chronic inflammatory demyelinating polyneuropathy (CIDP) with antibodies against NF155/contactin-1/CASPR1, 2) the skin and mucosa with skin blistering diseases such as pemphigus vulgaris (PV) and pemphigus foliaceus (PF), 3) the kidneys with PLA2R- and THSD7A- antibody positive membranous glomerulonephritis and 4) the haematological system with diseases such as thrombotic thrombocytopenic purpura (TTP, ADAMTS13) or GPIHBP1 autoantibody syndrome. Other unifying features of IgG4-AID are their low prevalence (10), their emerging strong genetic associations with specific HLA alleles such as HLA-DQB1*05 and HLA-DRB1*14 (18, 19), their clinical severity and chronicity and their good response to B cell depletion therapy.

How can the disease-overarching association of these common features be explained and which commonalities can we identify in the immune cells involved in the production of pathogenic IgG4 autoantibodies (2, 20)?

## IgG4-Isotype Antibodies: The Friendly Villain?

IgG is the main antibody class in the human body, with serum concentrations of 7-15g/L. However, the four IgG subclasses are numbered in descending order of their frequency, making IgG4 with an average of 5% (0.35-0.51g/L) of total IgG the rarest IgG subclass (21–23). Notably, IgG4 concentrations vary considerably between healthy individuals and throughout life (24), and may range from 0.01g/L to 1.4g/L (25), but may exceed these ranges in inflammatory conditions such as helminth infection to extreme levels such as 9g/L (26).

Normally IgG4 is produced after chronic exposure to antigen (27–31) or a strong antigenic stimulus such as in allergen immunotherapy (29–32), and it is usually induced by a class switch from IgE (or other antibody classes/subclasses) towards IgG4. IgG4 then competes with antibodies of other classes and subclasses for the antigen, and blocks the epitope so that the effector function of the competing antibody is abolished (30–38). Experiments using IgG4 derived from hyper-immune beekeepers show that it is protective in bee venom allergy patients (28, 39) and mice exposed to lethal africanised honey bee venom (40). IgG4 antibodies against the AChR protected experimental animals in a passive transfer model from the pathogenic effects of IgG1 antibodies against the AChR (20).

**Figure 1:**
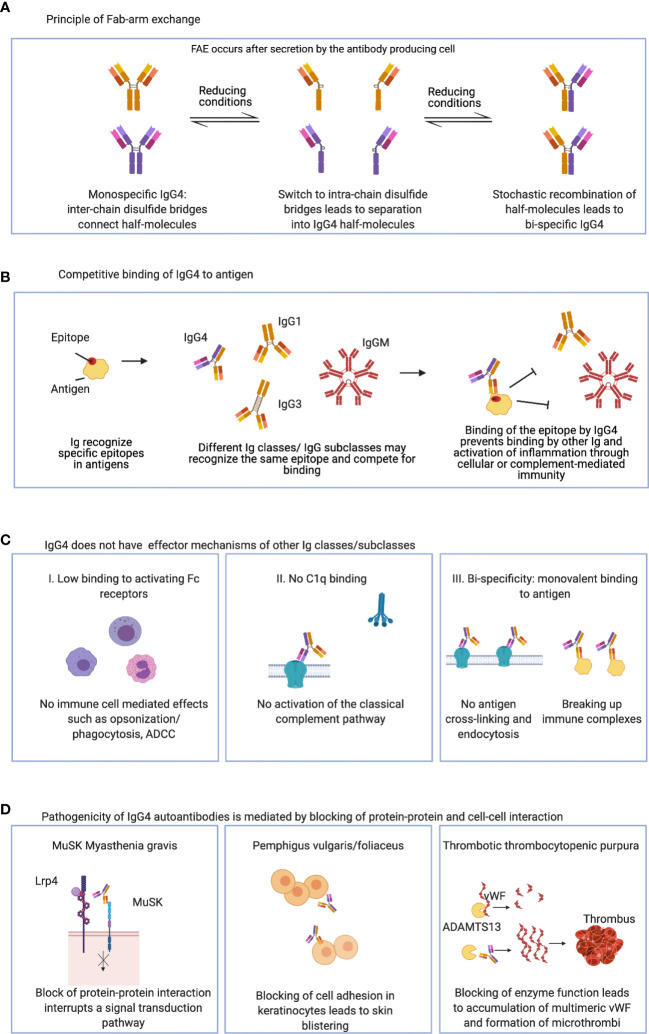
Charactristics of IgG4: **(A)** IgG4 can undergo Fab-arm exchange and form functionally monovalent antibodies. **(B)** IgG4 may bind and compete for binding of other Ig classes and IgG subclasses. **(C)** IgG4 has anti-inflammatory properties and lacks typical IgG effector mechanisms. **(D)** IgG4 autoantibodies can be pathogenic by direct blocking of protein-protein and cell-cell interaction. ADCC, Antibody-dependent cellular cytotoxicity; MuSK, muscle-specific kinase; Lrp4, low density lipoprotein receptor-related protein 4; vWF, von Willebrand factor; ADAMTS13, a disintegrin and metalloprotease with thrombospondin type 1 motif 13.

The four IgG subclasses share over 90% sequence homology, but single amino acid differences in IgG4 affect its structure and function substantially. IgG4 has anti-inflammatory properties (**Figure 1**), as it does not activate the complement system or engage activating Fcγ receptors on immune cells. A single amino acid difference in the hinge leads to increased stereometric flexibility and allows – under reducing conditions- the formation of intrachain disulphide bridges instead of interchain disulphide bridges, which leads to a loss of covalent connection of the two antibody half-molecules (41–43). This allows for a dissociation of IgG4 into two half-molecules that may then recombine randomly with IgG4 half-molecules of other specificities, and generate bi-specific functionally monovalent antibodies. This process is termed “Fab-arm exchange” (20, 44–50). Fab-arm exchange and bi-specificity were also observed in IgG4 autoantibodies against MuSK (50, 51). Functionally monovalent antibodies cannot cross-link antigen and induce antibody-induced antigen-internalization (**Figure 1**). Furthermore antigen-antibody immune complexes cannot be formed (20, 52, 53). Single amino acid differences in the CH2 region also render IgG4 immunologically inert: 1) a serine instead of a proline at position 331 (P331S) prevents binding of C1q and activation of the classical complement pathway (54, 55), while 2) further differences in the CH2 (L234F, P331S, A327G) reduce IgG4 binding to activating Fcγ receptors, which prevents activation of immune cells (56–61).

Therefore, IgG4 autoantibodies do not hold classical effector functions such as complement- or immune cell- mediated tissue damage. Rather they exert their effects by direct steric interference with their target antigens. Since they are unable to form immune complexes, this interaction would be beneficial in situation where non pathogenic antigen needs to be cleared and eliminated without causing overt inflammation. However, in the setting of autoimmunity, this mechanism has the potential to act as pharmacological antagonism at its target antigen. Increasing antibody concentrations will increasingly compete with physiological interaction of the targeted protein with its partners. And indeed, this has been found to be the main effect in many IgG4-AID such as MuSK-MG, pemphigus vulgaris or anti-LGI1 encephalitis (62–67). For example, antibodies to MuSK interrupt a signal transduction pathway required for the functioning of the neuromuscular junction, therefore causing muscle weakness in patients with MuSK-MG (62, 63), antibodies against desmoglein 1 and/or 3 lead to loss of cell adhesion between keratinocytes, therefore causing blistering of the skin in pemphigus patients (65, 68, 69), and inhibitory antibodies against the protease ADAMTS13 lead to an accumulation of multimeric von Willebrand factor and vascular occlusion in patients with TTP (70–72).

The autoantibody isotype and subclass is thus a critical factor in the pathomechanism of antibody-mediated autoimmunity. Yet it remains a crucial question, why IgG4 subclass autoantibodies are produced in the first place. What causes B-cells to undergo class-switching towards IgG4 and which cells are involved in its production?

## B Cells in IgG4-AID: The Gun That Shoots the Bullets

B cells are the antibody-producing cells of the adaptive immune system and are responsible for humoral immunity. B cells are also powerful antigen presenting cells and provide critical costimulatory signals to T cells. Naive B cells can develop into distinct cytokine-producing subsets that influence CD4+ T cell responses. Various cytokine-producing B cell subsets (TNF-α, CCL3, IFN-γ, GM-CSF, IL-6, IL-17, IL-2) have now been identified to modulate the polarization of CD4+ T cell responses *in vivo* (73, 74).

During isotype switching, which is required for the formation of immunoglobulin subtypes, T helper cells stimulate the progeny of IgM– and IgD–expressing B lymphocytes to produce antibodies of different heavy-chain isotypes. Heavy chain isotype switching is induced by a combination of CD40L-mediated signals and cytokines. These signals act on antigen stimulated B cells and induce switching in some of the progeny of these cells. In the absence of CD40 or CD40L, B cells secrete only IgM and fail to switch to other isotypes, indicating the essential role of this ligand-receptor pair in isotype switching. Activated B cells in germinal centers may differentiate into long-lived plasma cells or memory cells. The antibody-secreting cells, called plasmablasts, enter the circulation and migrate to the bone marrow or mucosal tissues, where they may survive for years as plasma cells and continue to produce high-affinity antibodies, even after the antigen is eliminated (75, 76). In anti-LGI1-encephalitis, cloning of recombinant human antibodies from the CSF of three patients indeed showed that 84% of all antibody-secreting cells and 21% of memory B cells did produce LGI1-specific antibodies (77).

Considering the substantial progress in B cell targeting therapy approaches in recent years, understanding the role of effector B cells and B cell dysregulation in subgroup specific differences of IgG4-AID is crucial. Although the proportion of B cells in the peripheral blood is not altered, memory B cells are increased in both AChR- and MuSK-MG patients (78). Notably, immunosuppressive treatment appears to reduce regulatory B cell subsets and increase memory B cell proportions in the peripheral blood (78–80). CD40 or TLR9-mediated IL-10 production of isolated B cells is reduced in MuSK-MG, suggesting suppressed regulatory B cell functions (78). Rituximab, a monoclonal anti-CD20 antibody, is highly effective in treatment of MuSK-MG and PV and appears to show its beneficial effects, at least partially, through repopulation of diminished IL-10 producing B cells (81). Although rituximab is also successfully used in other IgG1-3-AIDs, the general consensus is that IgG4-AIDs seem particularly sensitive and more often remain in long term remission (82–90). In a recent study, treatment with a low dose of teriflunomide was shown to ameliorate muscle weakness in the mouse model of MuSK-MG, predominantly through suppression of memory B cells in the lymph nodes without significantly reducing effector T cell populations (80). MuSK-MG patients also exhibit defective IL-6 production after B cell stimulation (78). This may be a compensating counter-measure to regulate autoantibody production, since IL-6 is involved in IgG class-switching (91).

The importance of B cells has been shown in other IgG4-AID as well. In great likeness to MuSK-MG, PV patients show increased memory B cell pools and impaired regulatory B cell functions. Treatment induced amelioration of PV symptoms is associated with reduction of memory B cells and enhancement of IL-10 producing regulatory B cells (92–94). Memory B cell levels are also correlated with disease activity and rituximab-mediated reduction of memory B cells result in amelioration of symptoms in TTP (95).

In brief, the dysbalance between memory/regulatory B cell activities appears to be a determining factor in IgG4-AID. Treatment response appears to be more closely associated with those targeting the aforementioned B cell subsets than other cell types of adaptive and innate immunity. However, what makes antigen-secreting B cells undergo class switch to IgG4? Does IgG4 have a physiological role and which cytokine factors are inductors of IgG4?

## Physiological Regulation of IgG4 Class Switch: A Path to Tolerance

Interestingly, IgE and IgG4 are both stimulated by the same Th2 cytokines, namely IL-4 and IL-13, and the determining factor, which induces the switch towards IgG4 production and IgE inhibition seems to be IL-10 (96–99). Among other cells, regulatory T cells (Tregs) secrete IL-10, which induces IgG4 production (100), and it is thought that this is mediated by signalling *via* glucocorticoid-induced tumour necrosis factor receptor-related protein (GITR) and GITR- ligand (GITR-L) (101). Regulatory B cells (Bregs) are mainly defined by their ability to secrete IL-10 *in vitro* (102–104). A common set of defining cell surface markers is not yet described, which may be due to the large variety of Breg subsets and the fact that every B cell subset has the ability to become an IL-10 producing Breg (103), even plasma cells (105). In one study, IL-10 producing Bregs were found to produce preferentially IgG4, in contrast to other B cell subsets (106). How exactly IL-10 promotes IgG4 and inhibits IgE production is not sufficiently understood. A recent study suggests that IL-10 directly increases the IL-4 induced IgG4 production in B cells over 20-fold, while the inhibition of IgE-production in B cells is indirect and requires the help of a further, yet unknown, cell population from PBMCs (107). In the context of allergy immunotherapy, the IgG4 response is also associated with a diversification and increased numbers of epitopes, while the number and diversity of IgE epitopes is reduced (108).

Taken together, IgG4 production is associated with anti-inflammatory tolerance mechanisms that is set in motion after prolonged or strong exposure to antigen and likely mediated *via* regulatory T and B cells *via* cytokines like IL-10 [discussed in more detail in (5)]. The induced switch towards the IgG4 subclass is thought to convey protection from chronic antigen exposure, including the potentially damaging effector mechanisms of other antibody classes and to save energy required to uphold a state of constant inflammation [**Figure 1** (30–38, 109)]. However, where does this tolerogenic mechanism get corrupted and skewed towards autoimmunity?

## Class Switch Towards IgG4 in Autoantibody-Associated Diseases: Good Intention, Bad Outcome?

One of the key questions is why the autoantibodies in IgG4-AID are predominantly of the IgG4 subclass. Indeed, it is currently unclear, why and how a preferential class switch towards the “protective” IgG4 subclass in patients with IgG4-AID is achieved and whether this is cause or consequence of disease chronicity.

A major challenge currently hampers our understanding of the class switch in IgG4-AID, which is the lack of commercially available and CE/FDA approved diagnostic tests that include autoantibody isotyping. The IgG subclass profiles of the autoantibodies are rarely analysed, and if so mostly in the context of basic or translational research studies. Quantitative IgG subclass profiles can be determined e.g. using flow cytometry (62, 82, 110, 111) or ELISA (112–114), which are research-based methods that are technically challenging, therefore not available for many diagnostic laboratories. The option to quantify antigen-specific IgG subclass profiles with certified, commercially available diagnostic test kits would greatly improve diagnosis and clinical management, and further both translational and clinical research into IgG4-AID, including the correlation between serological concentrations of the four IgG subclasses with clinical outcomes in response to different types of immunosuppression or the timing of the IgG4 class switch in the patients. With the data available to date, three different (but not per se mutually exclusive) scenarios can be hypothesized:

(1) After initial breach of tolerance and dominant relative low dose IgG1-3-mediated disease including complement mediated tissue damage, chronic antigenic exposure together with regulatory T-cell influence leads to class switch to IgG4. While this prevents complement mediated tissue damage, high-titer IgG4 autoantibodies cause direct steric interference with their target antigens and thus disrupt their respective function, which accelerates chronic ongoing disease activity. In other words, IgG1-3 autoantibodies may be of lesser dose or less pathogenic than IgG4 autoantibodies to the same target antigen. In most IgG4-AIDs, IgG4 levels predominate at time of diagnosis but often considerable levels of IgG1-3 antibodies against the same antigenic target can (still) be detected. Very recently, cloned recombinant human antibodies isolated from cerebrospinal fluid of three patients with the IgG4-AID anti-LGI1 encephalitis were analysed. In all three patients, IgG1, IgG2 and IgG4 LGI1 antibodies were identified (77). The class switch from IgG1/3 to IgG4 could merely be difficult to be observed, because by the time the disease has manifested and serum was tested for antibodies, the class switch had already taken place. Indeed, in some patients with IgG4-AID, class switch has been observed: In CNTN1 antibody-associated neuropathy and membranous nephropathy with PLA2R antibodies or acute autoimmune paranodopathy, a class switch from complement-fixing IgG1 or IgG3 in the acute disease phase towards IgG4 in the chronic phase has been observed (115–118). In TTP with anti-ADAMT13, a class switch to IgG4 has been documented before relapse in patients (119).

(2) Individual predisposition for antigen-specific autoimmunity mediated by certain MHC II- antigen- T-cell receptor combinations could predispose to a “skewed” isotype profile towards IgG4, as supported by the strong genetic associations with specific HLA alleles such as HLA-DQB1*05 and HLA-DRB1*14 (18, 19). This would mean, that IgG4-predominance occurs already early during the disease in predisposed individuals and causes chronicity rather than being a consequence of it: A recent study by Ellebrecht et al. (120) has looked into the evolution of autoreactive B cells in PV. B cells normally undergo class switch in the order IgM > IgG3 > IgG1 > IgA1 > IgG2 > IgG4 > IgE > IgA2, therefore it is possible that from the initial IgM class, first anti-Dsg B cells of a different antibody class or subclass (e.g. IgA) were generated before they switched to the IgG4 subclass. However, it was found that IgG4-specific B cells showed no clonal relationships to B cells with other subclasses and preferentially targeted desmoglein adhesion domains, while anti-Dsg B cells of other classes and subclasses also targeted other regions, suggesting that IgG4 anti-Dsg B cells evolve independent of other antibody classes and subclasses, perhaps by a direct switch from IgM to IgG4 (120). In line with this observation is the finding that IgG4, but not IgG1-3 antibodies against MuSK are able to block Lrp4-MuSK interaction, which suggests the recognition of different epitopes (62, 63). Another related possibility is a class switch first to IgG3, followed by an immediate switch to IgG4 due to unknown regulatory mechanisms. This possibility (and specifically IgG3) was not investigated in the Ellebrecht study (120), but another recent study suggested that a single amino acid replacement in IgG3, which increases its half-life, is associated with a risk to develop PV (121). This is interesting since the PV antibodies are not usually of the IgG3 subclass, but predominantly IgG4 and, to a lesser degree, IgG1 and IgA (122–125). Perhaps a switch to long-lived IgG3 leads to conditions where the immune system induces for yet unknown reasons an immediate and complete class switch towards IgG4. Still, one would expect remnant IgG3 to be present, yet in PV and MuSK MG this subclass is mostly absent in the antigen-specific IgG repertoire (62). Furthermore, some allergens e.g. food or tree pollen, seem especially inclined to induce IgG4 responses, as shown e.g. in response to banana, a lectin antigen (126) or increased IgG4 response to allergens in patients with IgG4-RLD (127).

(3) Aberrant B cell development in IgG4-AID may lead to individuals being more inclined to develop an IgG4 response. Serum IgG4 levels are generally normal or slightly elevated in IgG4-AID patients (14, 128, 129). Upon vaccination with a recall antigen, MuSK MG patients did not develop a disproportionately high IgG4 response (130). Moreover, circulating isotype-specific plasma cells and memory B cell numbers do not show large deviations from healthy individuals. Although many of these studies are challenged by the use of immunosuppressants by the patients, the data thus far does not suggests large B cell developmental problems in patients with IgG4-AID. Cells involved in regulation of the immune response do sometimes appear in abnormal levels. Especially lower Tregs and Breg numbers have been reported, but this is also found in other IgG1-IgG3 dominated AIDs.

Confirming or refuting one (or all) of these hypothesis is remarkably difficult in humans. Longitudinal analysis of patients starting at presymptomatic time points and including cell sampling from bone marrow and target organs would be required. Yet what translational insight can be gained from modelling IgG4-AID in model organisms? Are there suitable models and what are their limitations?

## Lessons Learned From Animal Models

Some evidence – albeit to some degree conflicting - can be gleaned from animal models. It is important to mention that mice do not have a proper equivalent of human IgG4, meaning mouse IgG subclasses do not undergo Fab-arm exchange and all have some immunogenic capacity. That being said, mouse IgG1 is the closest homolog to human IgG4, as it does not fix complement by the classical pathway and is driven by Th2 cells, which allows for studying the effect of vaccination for example in rodent models. Furthermore, mice have two IgG2 isotypes, IgG2a and IgG2b, although B6 mice, and a few other strains, lack the IgG2a isotype and have IgG2c instead. In B6 mice with the same genetic background, both AChR and MuSK antibodies can be induced with full-blown symptoms of myasthenic muscle weakness. While AChR autoantibodies are IgG2-dominant, MuSK antibodies are IgG1-(human IgG4 equivalent) dominant in B6 mice, suggesting that the direction of immune response is at least partially mediated by the molecular characteristics of the target antigen in addition to genetic factors (131, 132). Notably, both MuSK and Dsg-immunized mice show IgG1-dominant antibody responses in both sera and the target tissue (muscle and skin, respectively), already shortly after the immunizations. However, respectable levels of anti-MuSK and anti-Dsg IgG2 and IgG3 antibodies are also present in sera of immunized mice at the same time point, implying that class switch may occur from other IgG isotypes to mouse IgG1/human IgG4 in IgG4-AID or that these responses develop in parallel and are not clonally related (131–134). Moreover, in MuSK experimental autoimmune myasthenia gravis (EAMG) models, IgG1 KO mice are capable of developing pathogenic IgG2 and IgG3 antibodies and MuSK-binding IgG2+ and IgG3+ peripheral blood B cells, further conforming that autoantigens of the IgG4-AID may induce antibody responses of all IgG isotypes (131, 132). Significantly enhanced IgG3 responses in IgG1 KO mice suggests that mouse IgG1 (and putatively human IgG4) might be suppressing production of complement-fixing IgG isotypes and this might be an additional underestimated anti-inflammatory mechanism of the human IgG4 isotype (131, 132). In AChR-EAMG, IgG1 deficiency causes increased EAMG incidence and severity in parallel to enhanced AChR-specific IgG2 and IgG3 production further supporting this assertion (135). To the best of our knowledge, IgG1 KO experiments have not been conducted for other IgG4-AID and thus it is not certain whether the abovementioned results can be generalized to all autoantigens associated with IgG4-AID.

Passive transfer of MuSK-MG and PV IgG into experimental animals induce the characteristic clinical and pathological features of the human disease, indicating the pathogenic action of serum IgGs in both disorders. Passive transfer of equal amounts of patient derived IgG4 and IgG1-3 to mice also showed that IgG4 was pathogenic, while IgG1-3 were not (136). However, MuSK antibody levels in the IgG1-3 injected mice were below detection level, while they were clearly detectable in the IgG4-injected mice probably due to low amounts of MuSK-specific IgG1-3 within the IgG1-3 fraction and has to be interpreted with caution. Removal of anti-Dsg IgG by immunoadsorption abolishes the pathogenic activity of PV sera (137). Moreover transfer of splenocytes of Dsg-3-immunized mice to Rag-2 deficient mice, which are not capable of producing antigen-specific B cells, generated anti-Dsg-3 antibodies and induced PV in recipient mice (137). Similarly, administration of inhibitory monoclonal antibodies against human recombinant ADAMTS13, target antigen in TTP, resulted in reduced ADAMTS13 activity, severe thrombocytopenia and hemolytic anemia in baboons. Also, treatment of mice with anti-ADAMTS13 single-chain variable-region fragments generated fatal thrombocytopenia (138–140).

Immunization based-mouse models of PV show striking resemblance to MuSK-EAMG in terms of pathogenic factors and corroborate the significance of IL-4, IL-10 and IgG1 as major contributing factors of IgG4-AID pathogenesis. In the mouse model of PV, IL-4 and IL-10 producing Dsg-3-reactive T cells have been associated with pathogenicity and neutralization of IL-4 has led to suppression of anti-Dsg-3 production and reduced disease severity. Moreover, in the animal model of PV, IgG1 has been identified as the dominant isotype both in serum and on keratinocyte surfaces (134, 141, 142).

It is also possible that further antibody-mediated mechanisms may additionally contribute to disease. A relatively understudied common denominator might be antibody-mediated mitochondrial dysfunction and apoptotic cell death in IgG4-AID, which so far have been implicated in PV, membranous nephropathy/IgG4-related disease and anti-LGI1 encephalitis (143–147). For example, muscle samples of MuSK-MG patients often exhibit mitochondrial dysfunction. MuSK-immunized, but not AChR-immunized, mice show increased numbers of ragged red fibers (a histochemical sign of mitochondrial dysfunction) and decreased activity of mitochondrial enzymes indicating the role of MuSK-immunity in induction of mitochondrial dysfunction.

In this context, it should be considered that perhaps not all pathogenic effects in IgG4-AID are necessarily induced by directly antibody-mediated mechanisms. In resemblance to AChR-EAMG, where proinflammatory cytokines have been shown to induce muscle cell toxicity (148), non-antibody pathogenic factors causing IgG4-AID may also influence the functions of the target organs. For example, IL-4 has been shown to reduce ADAMTS13 expression thereby potentially contributing to pathogenesis of TTP (149).

The role of IgG4 antibodies is still unclear in anti-IgLON5 disease, where patients often show a rather “neurodegenerative” clinical phenotype, brain autopsies may present hyperphosphorylated tau-deposition (a marker of neurodegeneration) and anti-IgLON5 autoantibodies induce neurodegeneration *in vitro via* unknown intracellular mechanisms (150, 151).

Taken together, although it is possible that the production of IgG4-autoantibodies represents a “primary” phenomenon in IgG4-AID, and their ongoing production causes chronicity, for the time being, one cannot exclude that they are simply a “secondary” phenomenon and their ongoing production is a result of chronic antigenic “stimulation”. Hopefully, future systematic approaches using sophisticated single cell based high throughput directly *ex vivo* analysis of transcriptomes and B/T cell receptors of lymphocytes together with animal experiments will provide the definite answer.

Yet how do these insights from human and animal studies contribute to “personalized therapies”. Can IgG4-producing plasmablasts or downstream effects of IgG4 itself be targeted and what are potential side effects? Is the new subcategorization into IgG4-AID relevant from a therapeutical point of view or is their treatment identical to comparable IgG1-antibody mediated autoimmunopathies?

## Treatments of IgG4 Neurological Diseases

Overall, general or partially selective immunosuppression or immunomodulation is the major treatment applied to the autoimmune neurological diseases. Unfortunately, to date, there are almost no disease-specific therapies for neuroimmunological diseases. However, *in vitro* (cell cultures) and *in vivo* (animal, brain pathology and clinical) studies have suggested the presence of different pathophysiological mechanisms in patients with different antibody types and subtypes (152, 153). These findings suggest that special treatment requirements may apply to neuroimmunological diseases with different antibody subtypes, including patients with dominant IgG4 antibodies, and indeed IgG4-AID have a unique response pattern to conventional therapies for autoimmune diseases. Importantly, IgG4 is not the sole antibody present in IgG4-AID. IgG1 antibodies can be present, and may also contribute to disease. The patients are heterogenous and may react differently to treatments, perhaps due to the different involvement of immune cells. Distinct B-cell populations may predominate in different patients, IgG subclass profiles of autoantibodies may vary, which could explain the differences in clinical responsiveness towards different immunosuppressive drugs. As in most autoimmune conditions, steroids have - mostly empirically - been shown to be effective in IgG4-autoantibody mediated diseases. Most if not all treatment regimens at least initially contain corticosteroids (152, 154–157). Some physicians initially apply high-dose iv methylprednisolone and other start with oral steroids (1mg/kg bodyweight followed by tapering). However, in most instances, long-term steroids are needed as maintenance therapy to prevent relapses and oftentimes side-effects become a limiting factor. Even though many questions regarding steroid therapy in IgG4-antibody mediated diseases remain unclear, this review will focus on four specific therapies directly addressing IgG4 antibodies or their producing cells that we estimate to be the most relevant treatments across diseases.

### Plasmapheresis

Plasmapheresis (plasma exchange, PLEX) and Ig-apheresis remove the majority of antibodies located in the patient’s blood plasma [which does not extend to extravascular antibodies, which may account for up to 65% (158)]. Since autoantibodies directly cause disease, and are removed from the body, it is considered to provide immediate relief for patients and is utilized in cases of acute exacerbation. PLEX is the first line of treatment for TTP where it contributes significantly in survival of acute episodes of the disease (159), possibly not only by removing ADAMTS13 inhibitory antibodies but also by replenishing active ADAMTS13. In MG, PLEX has long been used as a treatment option, especially during myasthenic crises or pre-operatively (160). It is especially effective in MuSK-MG, probably more effective than in AChR-MG (161). Plasmapheresis has been successfully performed in pemphigus for over thirty years, particularly in cases of severe or recalcitrant disease, and as a steroid-sparing agent (162, 163). More recently, there is growing evidence to the therapeutic value of plasmapheresis in AE, including patients with GABA, LGI1 and AMPA antibodies. In a single center prospective study, significant improvement of the modified Rankin scale was seen in patients with severe refractory AE after treatment with PLEX, steroids and IVIG compared to those that only received steroids and IVIG (164). Studies have shown that both plasmapheresis and Ig-apheresis are well tolerated with minimal adverse effects supporting their use in clinical practice (165). Taken together, plasmapheresis appears to be a safe and efficient escalation therapy for acute IgG4-AID.

### IVIGs

Intravenous immunoglobulins (IVIGs) are pooled human IgG from many healthy donors, and as such comprised of a highly diverse antibody repertoire of human IgG1,2,3 and 4. IVIGs have a range of proposed mechanisms, of which some may be relevant and others irrelevant for IgG4-AID (166).

Possible mechanisms: A key mechanism of action of IVIGs may be the blocking of the neonatal Fc receptor (FcRn), which contributes to effective humoral immunity by recycling IgG and extending its half-life in the circulation to 3-4 weeks (167). IVIGs block and saturate these receptors, thereby increasing the degradation of IgG, reducing the half-life of IgG and ultimately leading to reduction of circulating IgG (168, 169) IgG1 and IgG4 both bind to the FcRn with similar efficiency (170), and blocking of the FcRn by a different drug led to reduction of both serum IgG1 and IgG4 (171). This mechanism of action is therefore available for IgG4 and may provide relief by enhancing IgG4 degradation.

In the highly diverse antibody repertoire of IVIGs, it is possible that antibodies against distinct autoantibodies, particularly against the antigen-binding part, termed anti-idiotype antibodies, can be present, and could neutralize the effects of autoantibodies (172–174). IVIGs may therefore also provide anti-idiotype antibodies that may bind to and neutralize pathogenic antibodies, but whether this applies to IgG4 autoantibodies is not clear.

Less likely mechanisms: Effect on proinflammatory cytokines: IVIGs may reduce the levels of proinflammatory cytokines that do not play a major role for IgG4-AID and induce anti-inflammatory cytokines (175–177). Effect on FcγRs: IgG4 has reduced binding affinity to activating FcγRs it does bind to FcγRIIB, which is an inhibitory IgG Fc receptor expressed on immune cells (170). IVIGs may bind and upregulate the receptors, therefore suppressing inflammation (178, 179), which is not essential in IgG4-AID. Furthermore, IVIGs have a range of inhibitory effects on other immune cells, e.g. monocytes, macrophages and dendritic cells (180–182).

Effect on complement: An important mechanism of IVIGs that is not relevant for IgG4 is the inhibition of complement activation, which is a main effector mechanism of IgG3 and IgG1, but not of IgG4 autoantibodies (183). Perhaps the degree of complement involvement in pathology is the determining factor for IVIG efficacy for the patients. In AChR-MG, complement-mediated damage is a key pathogenic mechanism, and IVIGs are considered as a short time treatment that provides immediate relief in AChR MG (160). In contrast, IVIG seems to be less efficient in MuSK MG, and particularly less effective than plasmapheresis (155), which could be due to the fact that MuSK IgG4 does not activate complement. A similar conclusion was drawn by Rodriguez et al. in a recent study with LGI1 encephalitis patients. In this large retrospective case series of 118 patients with LGI1 encephalitis patients, the authors observed that IVIGs were less efficient in comparison with corticosteroid treatment (184). Nevertheless, IVIGs did have an effect and fared better than placebo in a small randomized placebo-controlled trial, in which patients with anti-LGI1 antibody-associated epilepsy (all with predominantly IgG4 autoantibodies) showed response in 6 out of 8 patients in the IVIG group, compared to zero response in the 6 patients in the placebo group (185). In pemphigus, high dose IVIG treatment has been reported to be an efficient treatment in patients that are unresponsive to immunosuppression (186) most likely due to FcRn blocking by IVIGs and increased IgG degradation, as a rapid decrease of Dsg autoantibodies (IgG1 and IgG4) was observed following IVIg treatment (187).

While IVIGs are considered as short-term treatment for most IgG4-AID, it is also used as a long-term treatment in certain disorders such as inflammatory neuropathies (188). The underlying mechanism of action of IVIG in the treatment of CIDP remains unclear. Long-term IVIG treatment appears to decrease autoreactive CD4+ and CD8+ T effector memory cells (189) and enhance FcγRIIB expression on B cells thus reducing conversion of naïve B cells to memory B cells (176, 190).

Taken together, IVIGs have several potential mechanisms of action targeting different parts of the immune system, and depending on their respective role for disease, IVIGs may be more or less efficient. The effects on complement, cytokine production and cellular immunity may not have a major impact of IVIGs in most IgG4-AIDs. These anti-inflammatory actions may in theory also backfire in IgG4-AID: IVIGs upregulate regulatory T-cells (191, 192), which are known producers of IL-10, and induce production of IL-10 by macrophages (182). IL-10 is a regulator of IgG4 class switch and may as such even increase production of pathogenic IgG4. In contrast, FcRn blocking and increased degradation of IgG4 may provide therapeutic relief as e.g. observed in pemphigus.

### Rituximab

Targeting B-cells, which are the cells producing autoantibodies, is a main focus of therapy for IgG4-AID. CD20 is a marker that is expressed on B-cells throughout development, but not on the antibody-secreting cells, short-lived plasmablasts or long-lived plasma cells. Rituximab is an anti-CD20 monoclonal antibody and as such depletes B-cells expressing CD20, therefore the treatment efficacy of rituximab depends directly on the type of B-cells involved in the production of autoantibodies which also appears to be linked to the IgG subclass.

For example, in refractory AChR-MG, rituximab is usually the preferred treatment (193), but its efficacy is not conclusive, probably because AChR antibodies are secreted by long-lived plasma cells, e.g. residing in the thymus or bone marrow (194–196). In contrast, rituximab has a much more promising effect in the IgG4 associated form, MuSK MG where it was found to be very efficient (88, 197–201), leading to depletion of MuSK IgG4 (86). The generally accepted explanation to date is that the cells responsible for MuSK autoantibody secretion (such as plasmablasts) require constant replenishment by memory B cells that express CD20 and are therefore susceptible to rituximab treatment (202).

It has well been established that CIDP patients with neurofascin antibodies do not respond to steroid and IVIG treatment but to rituximab, which was found to enhance regulatory B cell responses and inhibit memory B cell and plasmablast populations (203). More importantly, rituximab suppresses short-lived plasma cells and their CD20+ precursors involved in IgG4 production (204). Rituximab is also considered as a very efficient treatment in TTP (205–207) and pemphigus (84, 85, 206, 208–212). Rituximab may be another efficient therapy in anti-LGI1 and CASPR2 encephalitis with predominantly IgG4 antibodies (213). Several studies suggest clinical usefulness of rituximab treatment for autoimmune encephalitis with antibodies against LGI1 and CASPR2, among these a large study from the GENERATE Registry with 62 patients with anti-LGI1 and 34 with anti-CASPR2 antibodies. The results suggest class IV evidence that early and short-term rituximab therapy could be an effective and safe treatment for these patients (214–216). An important limitation of these studies is that the IgG antibody subtype was not established. Future studies linking antigen-specific IgG-subtypes and specific immunotherapies are required to identify the best treatment for IgG4 of these patients.

However, because of its potential side effects and its high cost, in several countries rituximab is mostly used for the treatment of steroid-refractory MuSK-MG. In several cases, rituximab treatment was sufficient by itself, eliminating the need for other treatments or even leading to long term remission (201), and aggressive treatment strategies including rituximab led to better clinical outcomes of MuSK-MG patients in general (217). Clinical guidelines suggested the early use of rituximab when an initial standard immunosuppression treatment does not result in rapid remission (218). On another note, a study on cost of rituximab in patients with pemphigus vulgaris suggests that the initial higher cost of rituximab may be at least in part counterbalanced by reduced long-term treatment cost (219).

### Bortezomib

Several preclinical studies have shown a potential benefit from proteasome inhibitors, which target long-lived plasma cells, a subset of cells that remains untouched with all the therapies previously mentioned (220). Due to their high antibody production rate, plasma cells are highly sensitive to the inhibition of the ubiquitin-proteasome pathway, resulting in the accumulation of misfolded proteins, leading to plasma cell death. *In vitro* treatment of thymocytes from AChR-MG patients with bortezomib resulted in marked depletion of long-lived plasma cells and inhibition of autoantibody production (221), and treatment of animals (active immunization model for MG, EAMG) resulted in an improvement of the symptoms (222). The TAVAB clinical trial (ClinicalTrials.gov Identifier: NCT02102594), aiming to assess the efficacy of bortezomib, a first generation proteasome inhibitor, in generalized AChR-MG among other antibody-mediated autoimmune diseases was unfortunately prematurely terminated due to difficulties in recruitment (ClinicalTrials.gov Identifier: NCT02102594). Interestingly, a case report presented a therapeutic benefit of a patient with refractory MuSK-MG after treatment with bortezomib (treated in combination with rituximab, aciclovir and cotrimoxazole after initial courses of IVIG and prednisone) (223). Treatment with bortezomib has also shown to be efficient in refractory cases of autoimmune encephalitis (224–226). Its efficacy and safety is currently being tested in severe cases of NMDAR, LGI1 and Caspr2 autoimmune encephalitis, of which the LGI1 and Caspr2 antibodies have IgG4 as the predominant isotype (227). Among IgG4-AID, bortezomib was described as a good therapeutic option for CIDP patients (228) with severe relapses or an acute progression who did not respond adequately to regular immunosuppression treatment and rituximab (228). Additionally, in a patient with anti-pan-NF-associated neuropathy (predominantly of the IgG4 isotype) with severe clinical manifestations, it has also being shown reduction in antibody titer leading to clinical improvement after receiving a combination of IVIG, PLEX, rituximab and bortezomib (229). Bortezomib was beneficial in patients with relapsed/refractory TTP with an acceptable adverse event profile (230). Together with rituximab, bortezomib has shown to induce long-lasting remission in an acute refractory subgroup of patients with TTP (231). Additionally, some case reports of TTP patients unresponsive to plasma exchange, immunosuppressive therapy and rituximab, presented with a reduction in autoantibody levels and an improvement in clinical condition after treatment with bortezomib alone (232) or in combination with plasma exchange and steroids (233). The authors attributed the improvement to an abolition of residual autoreactive plasma cells. A new clinical trial to investigate the efficacy and safety of bortezomib as a first line treatment for acquired TTP has been approved, but at the last update in November 2021, recruitment had not yet begun (ClinicalTrials.gov Identifier: NCT05135442). Among the IgG4-RD spectrum, hyper IgG4 syndrome is characterized by the lymphoplasmacytic infiltration with IgG4-positive cells and a pronounced fibrosis of different tissues. Interestingly, a patient suffering from recurrent and unresponsive hyper IgG4 disease, in this case characterized by IgG4 plasma cell infiltration in the lungs, was successfully treated with a combination of bortezomib, dexamethasone and cyclophosphamide (234).

### Strategies for Novel and Specific Therapeutic Approaches

As described above, current treatments for IgG4- AIDs rely mostly on immunosuppression (corticosteroids or non-steroid immunosuppressants) and depletion of antibodies or B cell antigen-specific, which are non-specific. Therefore, they can be associated with side-effects, raising the need for novel, more targeted therapies.

Plasmapheresis or total IgG immunoadsorption is often beneficial in MuSK-MG, PV, TTP, CIDP, AIDP, Goodpasture’s syndrome and anti-DPPX encephalitis (235–242). A targeted approach would be the selective adsorption of the IgG4 or antigen-specific antibodies, while leaving unaffected the majority of plasma IgGs, thus avoiding hypogammaglobulinemia. In addition, the removal of a very small percentage of the total IgGs could prolong the treatment effect, due to the slower rate of autoantibody reappearance after treatment (243, 244). This could be accomplished by passing the plasma through a suitable matrix before being returned to the patient. In the case of MuSK-MG, we have explored an antigen-specific approach by the generation of sepharose-immobilized recombinant MuSK extracellular domains. The efficiency and specificity of the method has been established by *in vitro* experiments, where immunoadsorption resulted in almost complete removal of the autoantibodies from all the patients’ sera tested (245). In addition, *ex vivo* immunoadsorption in rats with EAMG, induced by immunization with human MuSK, resulted in a rapid and significant amelioration of symptoms, without the emergence of any adverse effects (246). Similarly, others have found that sepharose-immobilized Dsg-1 and 3 ectodomains successfully and specifically removed the Dsg antibodies and eliminated the pathogenic activity of PV patient sera (247). Although the demonstration of the proof of principle by these studies supports that similar strategies could be applied in other IgG4-associated diseases as well, it may be difficult and cost-prohibitive to optimize different matrices for each individual of these rare diseases. A similar but much more feasible solution could be the generation of matrices capable of binding only the IgG4 fraction of antibodies, thus removing the pathogenic autoantibodies, but not the majority of the IgGs (IgGs 1-3).

Another approach that gains ground as an antigen-specific therapy is the induction of tolerance towards the autoantigens, by administration of disease-relevant antigen derived domains and peptides. A recent study using MuSK-induced EAMG in mice demonstrated the potential for tolerance induction by oral administration of recombinant MuSK domains (248). However, MuSK administration took place prior to induction, so further studies are necessary to evaluate the actual therapeutic potential. Furthermore, it is not clear what IgG autoantibody subclasses were generated in the mouse model, so it is difficult to extrapolate the findings to IgG4-mediated MuSK-MG in humans. In fact, several lines of evidence support that tolerance induction works by diverting the immune response from a Th1 type to Th2/Th3 (249, 250). Furthermore, it appears that tolerance induction is accompanied by an increase in IL-10 levels, suggesting the diversion of the immune response towards IgG4 (251, 252). Therefore, it is still not clear how beneficial such an approach would be in the case of IgG4-associated autoimmune diseases, and further studies illuminating the underlying mechanisms are required.

IL-10 is essential in IgG4 class switch, however, it probably increases the production of IgG4 by enhancing IL-4 induced IgG4 switch (52, 99), making IL-4 also a key player in IgG4-RLD and, potentially, in IgG4-AID. Increased IL-4 levels have been associated with an increment in Th2 and plasmablast cell count and stimulation with IL-4 resulted in elevated IgG4 and IgG4:IgG ratios in patients with active untreated IgG4-RLD (253, 254), and lymph node cells and sera from MuSK immunized mice also showed upregulation of IL-4 and IL-10 (131).

**Figure 2:**
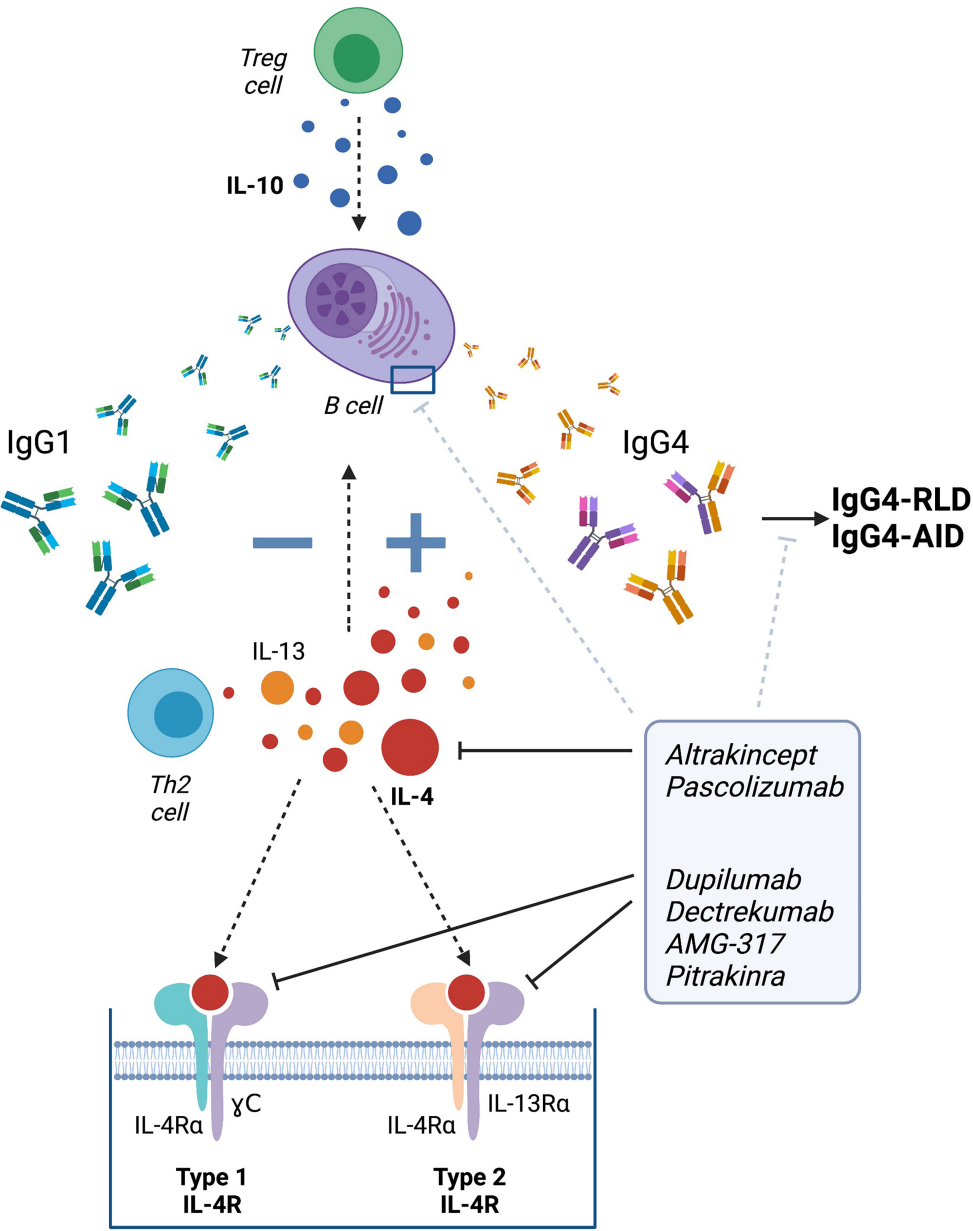
Therapeutic strategies targeting IgG4 class switch *via* IL-4. Altrakincept, pascolizumab, dupilumab, dectrekumab, AMG-317 and pitrakinra have been developed to prevent the binding of IL-4 to its receptor by blocking the soluble IL-4 or targeting its receptor itself. Targeting IL-4 may help to avert IgG4 class switch and therefore, the development of IgG4-RLD and IgG4-AID. Figure created using Biorender.

Several drugs targeting soluble IL-4 or its receptor have been developed (**Figure 2**). Among those, dupilumab is an IgG4 monoclonal antibody that acts on the IL-4 receptor alpha (IL-4Rα), a receptor that can bind to both IL-4 and IL-13 (255).

In IgG4-RLD, the efficacy of dupilumab has been described in a few case reports. After refusing treatment with other immunosuppressants, a patient with a complex and acute clinical picture including allergies, asthma, atopic dermatitis and a borderline diagnosis for IgG4-RLD was treated with dupilumab. After 12 months, the patient’s IgG and IgG4 levels decreased and most of the complaints resolved. Importantly, no relapses were observed and the patient did not suffer from any long-term side effects (256). Another case report described a patient with IgG4-RLD, who presented a good initial clinical response to steroid treatment. However, after suffering a relapse, rituximab was introduced, which helped initially to ameliorate disease, but could not prevent relapse one year later. Dupilumab treatment was started thereafter and led to rapid improvement accompanied by a biological reduction of all cells currently associated with IgG4-RLD (decrease of total T helper 2 cells, T follicular helper (Tfh) cells, Tfh2 cells and plasmablasts as well as serum IgE after 3 and 8 months of treatment). Interestingly, IgG4 levels remained stable. It is likely that the clinical improvement was not exclusively due to dupilumab treatment but perhaps due to combination therapy (with rituximab). The authors suggested that dupilumab could be a good treatment option for IgG4-RLD patients with relapses after an induction treatment (257). Another IL-4 targeting therapy known as dectrekumab (VAK-694), an IL-4 antagonist fully humanized monoclonal antibody, showed a decrease in the number of allergen-specific IL-4 producing cells in a clinical trial with patients with seasonal allergic rhinitis. However, VAK-694 did not show any extra benefit compared to patients treated only with the routine/standard therapy when looking at the symptoms (258).

Clinical trials are required to further investigate and validate the therapeutic benefit of anti-IL-4 therapies, but the initial data suggest that targeting the regulation of IgG4 production and IgG4 class switch *via* IL-4 may present a potential novel therapeutic strategy for IgG4-RLD, and perhaps also IgG4 autoimmune diseases (**Figure 2**).

## Conclusions

IgG4-AID affect different organs, are characterized by IgG4 autoantibodies targeting specific antigens and mostly display a strong genetic predisposition mediated by HLA-genes. IgG4-AID are distinct from IgG4-RLD which are characterized by fibrosis, elevated serum IgG4 concentrations and IgG4 positive plasma cell infiltrates in the affected tissue, but mostly lack antigen-specific IgG4. Hallmarks of IgG4 are the absence of complement activation, lack of bispecificity due to Fab arm exchange and inability to activate cells *via* Fc receptors. Instead, IgG4 autoantibodies are pathogenic by direct blocking mechanisms. Why and how IgG4 autoantibodies are produced is currently unknown, but we suspect that IL-4, IL-13 and IL-10 that are known regulators of IgG4 class switch may also play a role in the production of pathogenic IgG4, and a dysbalance between memory and regulatory B cells may determine the induction of IgG4-AID. Overall we propose three possible scenarios contributing to IgG4-AID immunopathogenesis: 1) initial disease includes antigen-specific IgG1-3 antibodies with low pathogenicity (e.g. *via* complement activation) or at low concentration, but chronic antigen exposure and influence from regulatory T cells induces class switch to IgG4, which then- either by increased dosis or pathogenicity- cause disease by steric hindrance. 2) Genetic predisposition, e.g. *via* distinct MHCII-antigen-T-cell receptor combinations may lead to a “skewed” isotype profile towards IgG4, or 3) aberrant B cell development in IgG4-AID may lead to individuals being more inclined to develop an IgG4 response. Further studies are necessary to investigate these different possibilities. Clinically, therapies targeting B cells have led to better clinical outcomes than general immunosuppression with corticosteroids in MuSK-MG and anti-LGI1 encephalitis, therefore we conclude that IgG4-AID may require different treatment strategies than classical antibody-mediated autoimmunity. We also discuss IgG4- or antigen-specific -apheresis and IL-4 receptor blockade as potential new treatment strategies for IgG4-AID. Finally, we think that the key to a better therapy lies in understanding the underlying immunopathogenesis and better diagnostics that also include the analysis of IgG subclasses to improve our assessment of treatment outcomes.

## Author Contributions

IK conceived the idea, wrote the manuscript, and provided figures. MM-D wrote the manuscript and provided a figure. VY, JT, KL, ST, ET and MH wrote the manuscript. IK, FL, RH, PM-M, and ET revised the final manuscript. All authors contributed to the article and approved the submitted version.

## Funding

IK was funded by a Hertha Firnberg Fellowship by the Austrian Science Fund, Austria, project number: T996-B30. FL is supported by German Ministry of Education and Research (01GM1908A) and E-Rare Joint Transnationalresearch support (ERA-Net, LE3064/2-1). JT and ST were cofinanced by the EU and Greek national funds through the Operational Program Competitiveness, Entrepreneurship and Innovation (project: T1EDK-05024, MIS 5032815). MM-D was funded by a Kootstra Talent Fellowship awarded by the Maastricht University Medical Center+. PM-M was funded by NIH 18-003826 and NIH 4224868 grants. MH receives financial support from the LUMC (Gisela Thier Fellowship 2021), Top Sector Life Sciences & Health to Samenwerkende Gezondheidsfondsen via the Target to B! Consortium (LSHM18055-SGF), PPS-Match call LUMC – Topsector LSH 2021, Prinses Beatrix Spierfonds (W.OR-19.13), and the Dutch Science Organization NWO (VENI 0915016181 0040).

## Conflict of Interest

JT and ST have shares in the research and diagnostic laboratory Tzartos NeuroDiagnostics, Athens. FL discloses having received speaker honoraria from Grifols, Teva, Biogen, Bayer, Roche, Novartis, Fresenius, travel funding from Merck, Grifols and Bayer and serving on advisory boards for Roche, Biogen and Alexion. MH Is a member of the European Reference Network for Rare Neuromuscular Diseases and The Netherlands Neuromuscular Center. MH is a co-inventor on two patent applications on MuSK-related research. LUMC and MH receive license income from these patents. LUMC receives royalties on a MuSK ELISA.

The remaining authors declare that the research was conducted in the absence of any commercial or financial relationships that could be construed as a potential conflict of interest.

## Publisher’s Note

All claims expressed in this article are solely those of the authors and do not necessarily represent those of their affiliated organizations, or those of the publisher, the editors and the reviewers. Any product that may be evaluated in this article, or claim that may be made by its manufacturer, is not guaranteed or endorsed by the publisher.

## Glossary

**Table:**

AChEi	Acetylcholinesterase inhibitors
AChR	Acetylcholine receptor
ADAM22	ADAM metallopeptidase domain 22
ADAMTS13	A disintegrin and metalloprotease with thrombospondin type 1 motif 13
ADCC	Antibody-dependent cellular cytotoxicity
AIDP	Acute inflammatory demyelinating polyneuropathy
AMPAR	α-amino-3-hydroxy-5-methyl-4-isoxazolepropionic acid receptor
Bregs	Regulatory B-cells
Caspr2	Contactin associated protein 2
CCL3	C-c motif chemokine ligand 3
CIDP	Chronic inflammatory demyelinating polyneuropathy
CNTN1	Contactin 1
DPPX	Dipeptidyl-peptidase–like protein-6
Dsg-1 Dsg-3	Desmoglein-1, desmoglein-3
EAMG	Experimental autoimmune myasthenia gravis
FAE	Fab-arm exchange
GABAaR	Gamma-aminobutyric acid a receptor
GABAbR	Gamma-aminobutyric acid b receptor
GITR	Glucocorticoid-induced tumour necrosis factor receptor-related protein
GITR-L	GITR- ligand
GM-CSF	Granulocyte macrophage colony-stimulating factor
HLA	Human lymphocyte antigen
IFN-γ	Interferon gamma
IgG4-AID	IgG4 autoimmune diseases
IgG4-RLD	IgG4 related disease
IgLON5	Immunoglobulin-like cell adhesion molecule 5
IL	Interleukin (IL-4
IL-10	IL-13)
IL-4Rα	Interleukin 4 receptor alpha
IVIG	Intravenous immunoglobulin
KO	Knock out
LGI1	Leucine rich glioma inactivated 1
LRP4	Low density lipoprotein receptor-related protein 4
MG	Myasthenia gravis
MN	Membranous nephropathy
MuSK	Muscle specific kinase
NMDAR	N-methyl-D-aspartate receptor
PBMC	Peripheral blood mononuclear cell
PF	Pemphigus foliaceus
PLA2R	Phospholipase A2 receptor
PLEX	Plasmapheresis or plasma exchange
PV	Pemphigus vulgaris
TNF-α	Tumor necrosis factor alpha
Tregs	Regulatory T cells
THSD7A	Thrombospondin type 1 domain containing 7a
TTP	Thrombotic thrombocytopenic purpura
vWF	Von Willebrand factor
